# Spatial and Temporal Characteristics of Hand-Foot-and-Mouth Disease and Its Response to Climate Factors in the Ili River Valley Region of China

**DOI:** 10.3390/ijerph18041954

**Published:** 2021-02-17

**Authors:** Suyan Yi, Hongwei Wang, Shengtian Yang, Ling Xie, Yibo Gao, Chen Ma

**Affiliations:** 1 College of Resources and Environmental Sciences, Xinjiang University, Urumqi 830046, China; yisuyan95@sina.com (S.Y.); xieling_1990@sina.com (L.X.); keioiobo@163.com (Y.G.); machen_666@163.com (C.M.); 2 Beijing Key Laboratory of Urban Hydrological Cycle and Sponge City Technology, College of Water Sciences, Beijing Normal University, Beijing 100875, China; yangshengtian@bnu.edu.cn

**Keywords:** hand-foot-and-mouth disease, spatiotemporal analysis, GAM, GTWR

## Abstract

Background: As the global climate changes, the number of cases of hand-foot-and-mouth disease (HFMD) is increasing year by year. This study comprehensively considers the association of time and space by analyzing the temporal and spatial distribution changes of HFMD in the Ili River Valley in terms of what climate factors could affect HFMD and in what way. Methods: HFMD cases were obtained from the National Public Health Science Data Center from 2013 to 2018. Monthly climate data, including average temperature (MAT), average relative humidity (MARH), average wind speed (MAWS), cumulative precipitation (MCP), and average air pressure (MAAP), were obtained from the National Meteorological Information Center. The temporal and spatial distribution characteristics of HFMD from 2013 to 2018 were obtained using kernel density estimation (KDE) and spatiotemporal scan statistics. A regression model of the incidence of HFMD and climate factors was established based on a geographically and temporally weighted regression (GTWR) model and a generalized additive model (GAM). Results: The KDE results show that the highest density was from north to south of the central region, gradually spreading to the whole region throughout the study period. Spatiotemporal cluster analysis revealed that clusters were distributed along the Ili and Gongnaisi river basins. The fitted curves of MAT and MARH were an inverted V-shape from February to August, and the fitted curves of MAAP and MAWS showed a U-shaped change and negative correlation from February to May. Among the individual climate factors, MCP coefficient values varied the most while MAWS values varied less from place to place. There was a partial similarity in the spatial distribution of coefficients for MARH and MAT, as evidenced by a significant degree of fit performance in the whole region. MCP showed a significant positive correlation in the range of 15–35 mm, and MAAP showed a positive correlation in the range of 925–945 hPa. HFMD incidence increased with MAT in the range of 15–23 °C, and the effective value of MAWS was in the range of 1.3–1.7 m/s, which was positively correlated with incidences of HFMD. Conclusions: HFMD incidence and climate factors were found to be spatiotemporally associated, and climate factors are mostly non-linearly associated with HFMD incidence.

## 1. Introduction

Hand-foot-and-mouth disease (HFMD) is a widespread intestinal epidemic caused by several enteroviruses that are characterized by fever and rashes or herpes on the hands, feet, and mouth. The first cases were diagnosed in New Zealand in 1957 [1], and HFMD has since become a global epidemic. In China, the first case of HFMD was confirmed in Shanghai in the 1980s [2], and it has been a major public health issue since 2007 when there was an outbreak in Linyi City, Shandong Province that resulted in 1149 cases with three fatalities [3]. China’s Ministry of Health established a national system to monitor HFMD in May 2008 [4], and, in 2014, HFMD became the most frequently reported monitored infectious disease [5]. In mainland China, there were approximately 1–3 million cases of HFMD from 2008 to 2019, which caused hundreds of deaths each year [6].

There is growing evidence that the global climate is changing rapidly, which highlights the need to examine the association between climate factors and infectious diseases [7]. Previous epidemiological studies have presented evidence of an association between climate factors and rates of HFMD [8,9]. In China, HFMD epidemics show seasonal variation. In southern cities, there are two peaks of HFMD, one in May and the other in October, while in northern cities, only one peak occurs annually in June [5]. The seasonality of HFMD indicates the potential role of climatic factors and demonstrates there are spatial and temporal variations of HFMD across China [10]. Several studies have reported that there is a non-linear relationship between climate factors, such as temperature and relative humidity, and the incidence of HFMD [11,12,13]. However, some studies have shown divergent conclusions, especially with respect to precipitation and wind speed. For example, a study in Guangzhou showed that for a 1 mm rainfall increase, it can positively affect the risk of HFMD [14], although this result was not proven in Shenzhen, China [15]. Wind speed was found to be positively correlated with the risk of HFMD in Beijing [16]. However, there was no such association between wind speed and the risk of HFMD in a study in Guangdong, China [17]. This may be because of differences in climatic factors and geographic conditions that lead to inconsistent results across studies. 

Although a potential relationship between the risk of HFMD and climate factors has been previously reported on a regional and national scale, this may not apply to arid regions due to spatial variation. The Ili Valley region is the only region in China influenced by the warm Atlantic Ocean current, and is a typical region of Xinjiang oasis, with the richest river in Xinjiang in terms of flow of water. The natural landscape of the Ili Valley region has distinct features, spanning temperate continental climate and alpine climate types. Its landform types are mainly divided into three types: mountains, hills and river valley plains. Under consideration of the natural geographical conditions of the Ili Valley, we first analyzed the spatiotemporal distribution of HFMD cases on a different scale, and next we quantified and characterized temporal and spatial variation between climate factors and the incidence of HFMD in township level with a geographical and temporal weighted regression (GTWR). Lastly, we used a Generalized Additive Model (GAM) to estimate the climate factor effect curve with the incidence of HFMD and explore the climate conditions suitable for the spread of HFMD.

## 2. Materials and methods

### 2.1. Study Area

The Ili River Valley is located west of the northern Tianshan Mountains in Xinjiang. The total land area is 5.6 × 104 km^2^, with a population of 26.39 million at the end of 2018 (Xinjiang Statistical Yearbook, 2019). The average annual temperature is 10.4 °C, the average annual precipitation is 417.6 mm, and the average annual amount of sunshine is 2870 h. The Ili River Valley region consists of eight counties (Yining, Nileke, Xinyuan, Gongliu, Tekesi, Zhaosu, Chabuchaer, and Huocheng) and two county-level cities (Yining and Huoerguosi) with eight subdistricts and 102 towns comprised of 24 Xinjiang Production and Construction Corps (XPCC) and 28 farms.

### 2.2. Data Collection

The data on HFMD cases for this article were obtained from the National Public Health Science Data Center. The number of cases, monthly incidences (per 100,000), and information comprising age, gender, occupation, address, and dates of onset and diagnosis were used to characterize the distribution of HFMD in the study area.

Climate data from 2013 to 2018 were obtained from the National Meteorological Information Center; monthly average temperature (MAT), monthly average relative humidity (MARH), monthly average wind speed (MAWS), monthly cumulative precipitation (MCP), and monthly average air pressure (MAAP) were analyzed.

### 2.3. Statistical Methods

Kernel density estimation (KDE) was used to identify the annual spatial distribution characteristics of HFMD in the study area. KDE is a non-parametric estimation technique that is widely used for spatial point pattern analysis and generates a smooth surface in a flat space reflecting continuous changes in the density of the point data [18]. A kernel is a circle with a predefined constant radius that is moved through the study area. The weight of each point depends on its distance from the center of the circle. A point near the center has a higher weight and vice versa [19]. We used the HFMD case data as point data input, and kernel density distribution was plotted for each year.

To further explore the differences in spatial and temporal distribution characteristics of HFMD, a retrospective temporal and spatial scan analysis was used. Scan statistics are widely used in disease surveillance and public health, not only to detect the spatiotemporal aggregation of diseases but also to identify areas of high risk for the onset of disease [20]. We used cities and counties as geographical units and months as the time scale based on the discrete Poisson model provided by SaTScan software. In terms of the model parameters, the upper limit of the clustering range was set to cover 50% of the regional population in the area, and the maximum time scan radius was set to six months. The difference in the number of incidences inside and outside of the scan window was regarded as statistically significant, as was the area that identified a high temporal and spatial clustering area of HFMD.

Geographically and temporally weighted regression (GTWR) was then applied to identify changing temporal and spatial associations between the incidence of HFMD and climate factors. GTWR embeds the time dimension into the regression model to measure the spatial and temporal variation of the data simultaneously and to better reflect the influence of the data between different adjacent temporal and spatial distances in the study area [21]. In this case, we used the spatiotemporal power function and the spatiotemporal distance of the Gaussian function proposed by Huang [22]. Initially, we needed to test for the multicollinearity of climate factors, so we set the climate factor to five independent variables with a monthly incidence as the dependent variable. We used the ArcGIS 10.5 GTWR analysis module and automatically optimized and set the bandwidth to 1 for the ratio of spatiotemporal distance parameters. The regression coefficients of influencing factors were then calculated and analyzed. The significance of the fit coefficient from the GTWR model was used to identify the association between each climate factor and the incidence of HFMD. A coefficient greater than 0 indicates a positive effect on the dependent variable and vice versa. The fitness of the model was conducted using the corrected Akaike information criterion (AICc) [23], adjusted coefficient of determination (*R*^2^), and residual sum of squares (RSS).

To quantify the effect of climate factors on HFMD, we assumed the Gaussian generalized additive model (GAM) distribution and simultaneously fit the model with a smooth curve for climate variables [24]. A GAM was applied during the multivariate analysis to evaluate linear and non-linear associations with climate factors, as the climate effect curve would be estimated in the entire study region. The fitness of the GAM was measured using generalized cross-validation (GCV) [25].

## 3. Results

### 3.1. Spatiotemporal Variation

#### 3.1.1. Year Scale Analysis

**Figure 1:**
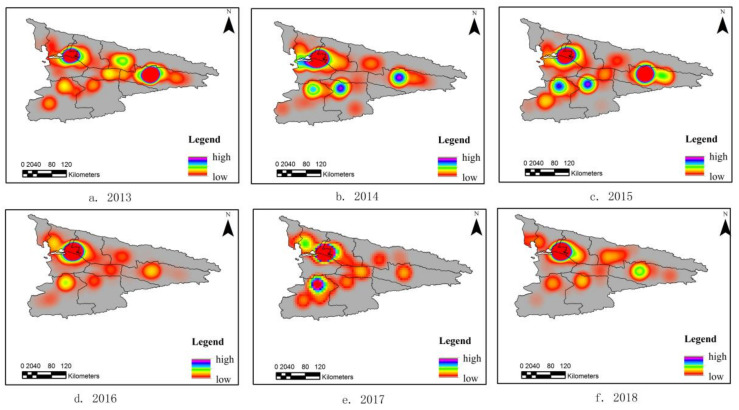
Kernel density maps for hand-foot-and-mouth disease (HFMD) from 2013 to 2018. ((**a**). 2013, (**b**). 2014, (**c**). 2015, (**d**). 2016, (**e**). 2017, (**f**). 2018).

Figure 1a–f display the KDE results that show that the highest density was centralized from north to south of the central region, gradually spreading to the whole region throughout the study period. The number of cases in the western plains area was higher than that in the eastern mountainous region. A high frequency of HFMD cases was also observed along the west of Yining City. From 2013 to 2015, HFMD incidences were spatially concentrated in the Ili Valley Basin region, and, after 2015, HFMD spread to a wide range of counties and cities, reaching its widest coverage in 2016.

In terms of kernel density values, there was a fluctuating trend in heat values from 2013 to 2015, the highest of which was in 2014. The disease incidence area was relatively concentrated, and the general trend showed a small range and high intensity. In 2015, four major agglomerations were formed in Yining City, Xinyuan County, Zhaosu County, and Tekesi County. From 2016 to 2018, the heat value showed a fluctuating trend, reaching its lowest in 2017.

#### 3.1.2. Month Scale Analysis

**Figure 2:**
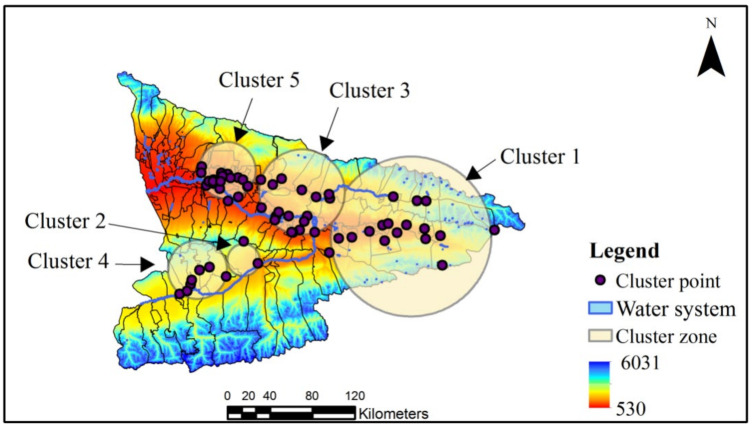
Locations of the space–time clusters in the study area.

Space–time cluster analysis using SaTScan identified five space–time clusters of HFMD cases in the Ili River Valley region from 2013 to 2018 (Table 1, Figure 2), and there was a difference between the results and the HFMD annual variation based on kernel density analysis. The most likely cluster was seen in the northeastern district from April to September 2013. Another cluster was seen in two southwestern districts, clusters three and four, from May to October 2018. A significant spatiotemporal cluster was detected south of Tekesi city from February to April 2016. The results show that five potential clusters were distributed along the Ili and Gongnaisi river basins.

### 3.2. Temporal and Spatial Variation between Climate Factors and the Incidence of HFMD

#### 3.2.1. Regression Model Selection

Table 2 shows that the results of the variance inflation factor (VIF) values of all variables were less than 10, so it could be assumed there was no multicollinearity between the variables, and all selected variables were involved in the model.

Table 3 summarizes the accuracy of the GTWR model, which was verified by taking the goodness-of-fit (*R*^2^), RSS, and the modified AICc value for the three types of models. The higher the *R*^2^ value, the smaller the AICc and RSS values, indicating that the independent variable was more explanatory than the dependent variable. The GTWR was selected as it had the smallest AICc value and highest *R*^2^ value. Further results are from the GTWR.

Table 4 shows the results of the GAM analysis. The variance inflation factor (VIF) values of all variables were less than 10, so it could be assumed that there was no multicollinearity between the variables, and all selected variables were involved in the model. The results of the smooth function test of MCP, MAAP, MAT, and MAWS were statistically significant, and the MARH was not statistically significant. For the model fitting results, *R*^2^ was 0.474, the variance explanation rate was 52.6%, and the GCV value was 8.8745. Additionally, the intercept is 2.20471, significantly correlated at the *p* = 0.001 level. The influence of the explanatory variables was measured by the magnitude of the F value, which shows that F (MAT) > F (MAWS) > F (MCP) > F (MAAP), where the F value of MAT had a larger variance than the other three climate factors. This indicates that MAT was the most important climate factor influencing the incidence of HFMD.

#### 3.2.2. Temporal Distribution

**Figure 3:**
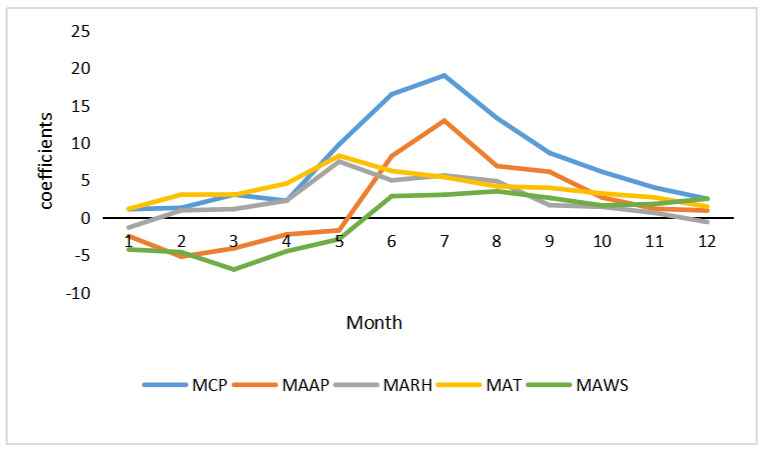
Temporal variations of the coefficients for climate factors.

The results from Table 5 demonstrated obvious spatiotemporal non-stationarity in the GTWR model. The average values of the coefficients between single climate factors and HFMD in the time dimension are shown in Figure 3, with the folded line representing the effect of different months on the incidence rate of HFMD for each variable.

From February to September, the incidence rate of HFMD varied significantly according to climate factors, with a positive correlation of MCP, MAT, and MARH. From February to August, the MAT and MARH fit curves showed an inverted V-shape. As temperature and humidity increased, the incidence rate of the disease showed an upward trend, with the effect being most pronounced in May. From January to May, the fitted curves of MAAP and MAWS showed a U-shaped change and a negative correlation with the rate of HFMD. The relationship between climate factors and HFMD showed fluctuating changes in spring, more significant changes in summer, and it gradually weakened in autumn and winter. The change in the fitted MCP curve was consistent with the annual precipitation pattern of the region, which is four consecutive months from April to July; the fitted curve showed an increasing trend, indicating that precipitation during this period contributed to the spread of HFMD. The MAT coefficient was positively correlated with an inverted V-shaped curve, and the incidence rate showed an increasing trend with increasing temperature; this was most significant in May. The MAT fitted curve was consistent with the change in the MARH curve, probably because HFMD is more active in humid and hot environmental conditions. The seasonal variation of wind speed in this region is characterized by maximum wind speed in spring, then summer, and the minimum is in winter. The MAWS coefficients varied more significantly, but the overall fluctuation was characterized by change.

#### 3.2.3. Spatial Distribution 

We applied ArcGIS to visualize the spatial heterogeneity of the mean of the coefficients and used 0 as an investigative value for positive and negative effects. For the test of spatial non-stationarity on the residual of the model result, the z-value is −0.65, which denotes 10% levels of significance. This means that the residuals are distributed randomly, indicating a strong regression and reliability of the model. Among the individual climate factors, the MCP coefficient values varied the most while the MAWS values varied less from place to place. There was a partial similarity in the spatial distribution of the coefficients for MARH and MAT, as evidenced by a significant degree of fit performance in the whole region.

**Figure 4:**
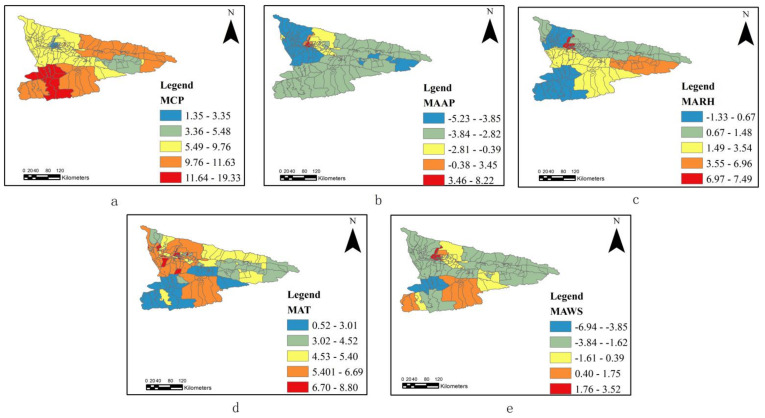
Spatial distribution of the coefficients for climate factors. ((**a**). MCP, (**b**). MAAP, (**c**). MARH, (**d**). MAT, (**e**). MAWS).

Figure 4a shows that the coefficient for MCP was positively correlated with HFMD. The natural terrain of the Ili River Valley opens up to the west in a trumpet shape so that humid airflow from the west easily enters the basin and, at the same time, the mountains in the southeast intercept the topographic precipitation in the mountains. Precipitation characteristics are higher in the east than the west and are more pronounced in the mountains than the plains, with windward slopes greater than leeward slopes. The areas with the highest coefficients are located in the southern region of the Tekesi–Zhaosu basin and the northeastern region of the Kashgar River hills. These areas are on the windward side, creating favorable conditions for the formation of topographic rain, whereas the central and western parts of the region have relatively low precipitation. Based on the results, we hypothesized that, in areas with abundant precipitation, viruses may spread using aerosols. Figure 4b indicates that the MAAP coefficients were more variable in the central region and less variable in the eastern region, which may be because the central regions are at a lower altitude. The coefficient decreases with increasing altitude. Figure 4c shows a decrease in the geographic variation of the MARH coefficients from a positive to a negative correlation from the central plains to the mountainous area. This indicates that the complexity of climate factors may affect the development of infectious diseases, and there may be an interaction between relative humidity and other climate factors on the occurrence of HFMD. Figure 4d shows the MAT coefficients affecting the northwest and central regions more significantly. Temperatures in the region had distinct horizontal and vertical zonalities with more complex vertical variations. The northwestern and central plains are at lower elevations compared to the southeastern mountains, and the enclosed land around the mountains gives the region higher than average temperatures, which are associated with HFMD. A small area in the southeast has a positive correlation between temperature and HFMD, probably because the area is in the temperature inversion zone. The positive correlation between the coefficients at high temperature and low humidity and the negative correlation between the coefficients at low temperature and high humidity may be because viruses are more likely to spread in a high temperature and low humidity environment. 

The coefficient analysis of MAWS in Figure 4e shows significant spatial differences. The topography of the Ili River Valley region shows that the mountainous areas are more affected by the prevailing westerly wind belt than the plains. Based on these results, we hypothesized that there may be a correlation between the spread of HFMD and wind speed.

The results above demonstrate differences in the main distribution intervals of each climate factor corresponding to positive and negative influences. However, there is still an obvious cross-sectional interval, indicating that the fitted values of climate factors were not the single determinant of incidence rate and need to be further analyzed in terms of the relationship between the temporal and spatial distribution of multiple climatic factors and incidence rate.

### 3.3. Quantification of Climate Factors Affecting the Incidence of HFMD

**Figure 5:**
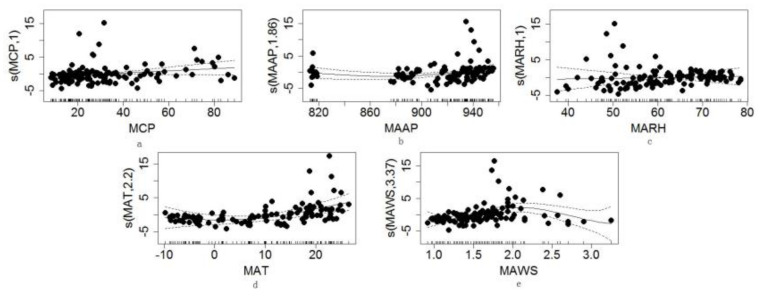
Effect of multifactorial climatic factors on the variation of HFMD concentrations. ((**a**). MCP, (**b**). MAAP, (**c**). MARH, (**d**). MAT, (**e**). MAWS)

The curve in Figure 5 is a smooth function of each climatic factor, and the vertical axis shows the effect of each factor on HFMD incidence. Multifactorial correlation analysis showed that MCP was positively correlated with HFMD incidence. Figure 5a shows an increase in the incidence of HFMD when MCP is within the range of 15–35 mm; when it exceeds 35 mm, the incidences gradually increase but at a slower rate. Figure 5b displays the MAAP results in a substantially inverted J-shaped curve because when MAAP was in the range of 925–945 hPa, it was positively correlated with HFMD incidence. However, in the range of 810–820 hPa, it was negatively correlated with HFMD incidence. As shown in Figure 5c, there was no correlation between MARH and HFMD under the combined effect of multiple factors. MAT values form a U-shaped curve, as shown in Figure 5d, with the incidence of HFMD gradually increasing with increasing temperature; the positive correlation was most significant in the range of 15–23 °C. MAWS had a roughly inverted U-shaped curve in the range of 1.3–1.7 m/s (Figure 5e), which showed a significant positive correlation with HFMD incidence. As wind speed picked up, it showed a negative correlation with the incidence of HFMD, i.e., at more than 2.0 m/s.

## 4. Discussion

In this paper, we initially analyzed the spatial and temporal distribution characteristics of HFMD through two different time scales. We then used the GTWR model to identify temporal and spatial changes in the associations between the incidence of HFMD and climate factors. We also used GAM to qualify the multiple climate factors affecting HFMD incidence. The associations were smaller in magnitude than those observed in other studies [26,27]. 

Our results indicate that the incidence of HFMD has regional differences and seasonal trends and that the incidence is higher in the plains than in the mountains. Spatial distribution was evident along the Ili and Gongnaisi river basins, probably because the plains are more residential and have a high population density, which can lead to clustered infection. This is supported by a study in Chongqing that found the location of clustering centers was concentrated in urban areas [28]. Our study demonstrates a non-linear relationship between climatic factors and the incidence of HFMD in the Ili River Valley region. We also found that the association between temperature and the incidence of HFMD presented an inverted V-shape with a positive correlation. These findings are consistent with a previous study in Wuhan, China [13].

Other results of our study show that MARH was positively correlated with temporal variations in HFMD incidence, but there were differences in spatial distribution. There were also indications that there may be differences in the influence of relative humidity on the spread of HFMD at different levels, that climate factors have a complex mechanism on HFMD occurrence and the development of infectious diseases, and that there may be some interaction between relative humidity and other climate factors on the occurrence of HFMD. Studies have shown that enteroviruses are more prone to transmission in high-humidity environments [29,30], which is supported by laboratory experiments that have found that viruses survive longer in humid environments than in dry air [31].

We found that MCP had a positive correlation with the incidence of HFMD, which is consistent with previous studies in Hefei, Shenzhen [32,33]. Moreover, high precipitation could promote virus attachment to small particles in the air or toys and food, thus increasing the risk of HFMD [28]. Heavy precipitation also increases the risk of water supply contamination from sewer overflows, so extreme precipitation may increase enterovirus exposure levels, leading to an increased incidence of HFMD [34].

Stronger wind speeds may lead to a higher probability of HFMD infection via respiration. This is supported by a study in Hong Kong, which found that, in developed areas, HFMD is more easily transmitted by respiratory droplets than by the fecal–oral route [35]. However, two studies from Shanghai and Hong Kong found a negative effect of wind speed on the incidence of HFMD [36,37]. Another study found that higher wind speeds significantly diminished the number of infectious airborne particles, eventually leading to a statistically significant reduction in exposure to infectious particles [38]. These different findings emphasize the necessity for more research on this topic. One possible reason for inconsistent results is the differences in geographic location, climatic environment, and socioeconomic conditions in each study area. Moreover, the choice of different data sources and weather variables may also lead to differences in the results [39].

In our study, we found that the association between MAAP and the incidence of HFMD may be related to the shift in winter and summer air pressure in the region. The entire Ili River basin is controlled by Mongolian high pressure in winter, and the area south of the Tianshan Mountains is controlled by South Asian subtropical low pressure in summer. There were significant differences in seasonal variation, and when we quantified the effect of air pressure on HFMD incidence, we obtained the same result. We hypothesized that high pressure was negatively correlated with the incidence of HFMD but that low pressure could facilitate the spread of HFMD. One study in Guangdong revealed a 6.8% drop in cases for every 1 hPa increase in air pressure [40]. This may be because lower air pressure may weaken the human’s/organism’s immune system [41]. However, for the spatial distribution of coefficients between the incidence of HFMD and MAAP (Figure 4b), we speculated that the variation in air pressure might be influenced more by altitude or factors other than air pressure.

Climate factors can potentially affect the incidence of HFMD either through affecting the reproduction and activity of the enterovirus or by affecting the activities and communication methods of people. Furthermore, HFMD epidemiological characteristics may be determined by the wet, hot weather in summer, as hot and humid conditions are suitable for HFMD pathogens to survive, spread and, subsequently, infect humans. The dry weather in autumn may increase the desire to travel, further increasing human-to-human communication and promoting the spread of HFMD. Under the combined influence of multiple climate factors, there was no correlation between mean relative humidity and the incidence of HFMD, which may be due to regional differences in the natural environment of the study area, which was located in a typical arid oasis with low humidity. In recent years, the Ili River Valley climate has tended to be warmer and dryer, so, in this study, the average relative humidity had a weaker effect on the incidence of HFMD and needed to work synergistically with other factors. These inconsistent results may be due to the different local climatic conditions, demographic characteristics, and socioeconomic factors of different regions.

There were previous studies conducted in exploring spatiotemporal changes of HFMD and the effects of climate factors on this disease [27,42]. Regression models were commonly used to evaluate the relationships between possible influencing factors and the number of HFMD epidemic cases [43,44]. Regression parameters were utilized as functions to describe the spatial and temporal position of sample data in the GTWR models [45]. The calculation accuracy of the GTWR model was higher than that of the two models because spatial and temporal weights in these functions can better respond to the influencing factors in different spatial and temporal locations.

This study had some limitations. First, we used average climate factor values as independent variables, which may result in misclassification bias. Second, the model generally shows some uncertainties, which could affect the final assessment result. Third, we analyzed the association between the spatial and temporal distribution of infectious diseases and meteorological factors, but the spread of disease is influenced by a variety of factors. The possible pathogenesis has not been considered from a socioeconomic, behavioral, or physiological perspective.

## 5. Conclusions

This study confirms that the spatial and temporal distribution of HFMD varies at different scales. The results show that the incidence of HFMD had regional differences and seasonal trends, and the incidence was higher in plain areas compared to mountainous areas, with a clear spatial distribution in the Ili and Kungnese river basins. 

The GTWR model results show that the incidence of HFMD and climate factors was found to be spatiotemporally associated. In the GAM model with multiple meteorological variables, climate factors are mostly non-linearly associated with HFMD incidence. Conducted to identify the climate conditions suitable for the spread of HFMD and to provide a reference for the prevention and control of HFMD. Further research in areas with more climate variability, such as the Ili River Valley region, could provide us with a better depiction of spatiotemporal variation.

## Figures and Tables

**Table 1:** Statistical results of the HFMD retrospective space–time scan.

No.	Coordinates	Locations	Radius ^1^	Time	No. of Cases	No. Expect	LLR ^2^
1	(43.423871 N, 83.521495 E)	Alemale Town, Tuergen Town, Areletuobie Town, Xinyuan Town, Biesituobie Town, Wugong Town, 71 XPCC, Zeketai Town, Kaisu Town, Breeding Bee Farm, Talede Town, 72XPCC, Xiaoerbulake Town, Wulasitai Town, Nalati Town, Musi Town, Kuerdening Town, Kalabula Town	75.49	April 2013–June 2013	163	33.59	129.80
2	(43.205440 N, 81.665532 E)	Qilewuzike Town, Tekesi Town	15.18	February 2016–April 2016	140	27	53.32
3	(43.999270 N, 81.480720 E)	Tulufanyuzi Town, Panjing Town, Sadikeyuzi Town, Kalayageqi Town, agricultural center, Kaerdun Town, Dadatumu Town, Alanmubage subdistrict, Tashikeruike Town, Jiefang subdistrict, Qiongkeruike subdistrict, Doulaitibage subdistrict, Tuogelake subdistrict, Dunmaili subdistrict, Quluhai, Town, 70 XPCC, Sabuyi subdistrict, Yuqunweng Hui Town, Kazanqi subdistrict, economic cooperation zone, Yili dairy farm, Hanbin Town, Awuliya Town, Bayandai Town, Kebokeyu Town, Yilihe subdistrict, Miquan Hui Town, Arewusitang Town, Youth farm, Yingyeer Town, Kuohongqi Town	26.90	May 2018–October 2018	1048	798	46.91
4	(43.157293 N, 81.130975 E)	77 XPCC, Hongnahai Town, Zhaosu Town, Ambanbagh Breeding farm, Wuzunbulake farm, Akedala Town	27.77	August 2018–September 2018	44	11	28
5	(43.808751 N, 82.356540 E)	Keling Town, Jiahawulasi Town, Wuzan Town, Nileke Town, Kalatuobie Town, Subutai Town, Hujier Town, Kolkhoot Haor Mongolian Town, Mazha Town, Agaersen Town, Kosh Agash sheep farm, cow farm, Kashi Town, 73 XPCC, Aketubieke Town, Gongliu Town, Liangfan field, Dunmazha Town, Samuyuzi Town, Hudiyuzi Town	40.27	August 2013–September 2013	19	3.75	15

^1^ Size of cluster in kilometers; ^2^ Log-likelihood ratio: risk within the scanning window compared to outside each location at *p* = 0.001.

**Table 2:** Result of colinearity statistics.

Independent Variable	MCP	MAAP	MARH	MAT	MAWS
VIF	1.559	1.366	6.407	5.712	1.851
Tolerance	0.626	0.732	0.256	0.375	0.540

**Table 3:** Comparison of the results of ordinary least square (OLS), geographically weighted regression (GWR), and geographically and temporally weighted regression (GTWR) in models *R*^2^, corrected Akaike information criterion (AICc), and residual sum of squares (RSS).

Model	*R* ^2^	AIC_C_	RSS
OLS	0.27	618.85	1104.03
GWR	0.28	622.91	1102.77
GTWR	0.52	610.99	736.19

**Table 4:** Statistical characteristics of variables and GAM fitting results.

Independent Variable	Edf ^1^	Ref.df ^2^	F ^3^	*p*-Value ^4^
MCP	1.00	1.000	6.169	0.014507
MAAP	1.860	2.083	3.208	0.042133
MARH	1.000	1.000	0.181	0.671396
MAT	2.049	2.310	7.763	0.000575
MAWS	1.936	1.996	6.317	0.003371

^1^ Edf: effective degrees of freedom; ^2^ Ref.df: reference degrees of freedom; ^3^ F: F-test; ^4^ Risk within the scanning window compared to outside each location at *p* = 0.001.

**Table 5:** Non-stationarity of parameters in the geographically and temporally weighted regression (GTWR) models.

Explanatory Variables	F	*p*-Value
MCP	4.150	0.004
MAAP	4.286	0.004
MARH	3.995	0.005
MAT	9.934	0.000
MAWS	7.008	0.000

## Data Availability

3rd Party data, Not applicable.
